# Anti-inflammatory effects of low-level laser therapy on human periodontal ligament cells: in vitro study

**DOI:** 10.1007/s10103-017-2376-6

**Published:** 2017-11-07

**Authors:** Ji-Hua Lee, Min-Hsuan Chiang, Ping-Ho Chen, Mei-Ling Ho, Huey-Er Lee, Yan-Hsiung Wang

**Affiliations:** 1 School of Dentistry, College of Dental Medicine, Kaohsiung Medical University, 100, Shih-Chuan 1st Road, Kaohsiung, 80708 Taiwan; 2 Orthopaedic Research Center, College of Medicine, Kaohsiung Medical University, Kaohsiung, Taiwan; 3 Institute of Biomedical Sciences, National Sun Yat-sen University, Kaohsiung, Taiwan; 4 Cancer Center, Kaohsiung Medical University Hospital, Kaohsiung Medical University, Kaohsiung, Taiwan; 5 Department of Physiology, College of Medicine, Kaohsiung Medical University, Kaohsiung, Taiwan; 6 Department of Marine Biotechnology and Resources, National Sun Yat-Sen University, Kaohsiung, Taiwan; 7 Department of Dentistry, Kaohsiung Medical University Hospital, Kaohsiung Medical University, Kaohsiung, Taiwan

**Keywords:** Low-level light therapy, Cyclic AMP, NF-kappa B, Cytokines, Interleukins, Lipopolysaccharides

## Abstract

Periodontal disease is a chronic inflammatory disease that is commonly treated with surgical and nonsurgical techniques. However, both approaches have limitations. Low-level laser therapy (LLLT) has been widely applied in reducing inflammatory reactions, and research indicates that LLLT induces an anti-inflammatory effect that may enhance periodontal disease therapy. The purpose of this study was to investigate the anti-inflammatory effect of LLLT on human periodontal ligament cells (hPDLCs) in an inflammatory environment and aimed to determine the possible mechanism of action. Cells were cultured and treated with or without lipopolysaccharide (LPS) from *Porphryromonas gingivalis* or *Escherichia coli*, followed by irradiation with a gallium-aluminum-arsenide (GaAlAs) laser (660 nm) at an energy density of 8 J/cm^2^. Quantitative real-time polymerase chain reactions were used to assess the expression of pro-inflammatory genes, including tumor necrosis factor-α (TNF-α), interleukin (IL)-1β, IL-6, and IL-8. The dual-luciferase reporter assay was used to examine nuclear factor-κB (NF-κB) transcriptional activity. An enzyme-linked immunosorbent assay was used to monitor the concentration of intracellular cyclic adenosine monophosphate (cAMP). Both LPS treatments significantly induced the mRNA expression of pro-inflammatory cytokines. However, LLLT inhibited the LPS-induced pro-inflammatory cytokine expression and elevated intracellular levels of cAMP. The LLLT inhibitory effect may function by downregulating NF-κB transcriptional activity and by increasing the intracellular levels of cAMP. LLLT might inhibit LPS-induced inflammation in hPDLCs through cAMP/NF-κB regulation. These results should be further studied to improve periodontal therapy.

## Introduction

Periodontal disease is a common and complex inflammatory disease that results in progressive destruction of the tooth-supporting soft and hard tissues of the periodontium [1]. The resulting damage affects the alveolar bone as well as the periodontal ligament (PDL). Although surgical therapies such as open flap surgery and osseous surgery and nonsurgical therapies such as standard oral hygiene procedure and sustained local delivery antimicrobial agents are helpful in controlling this disease, both treatment options have limitations. Patients with deep pockets or furcation involvement did not respond well to nonsurgical therapy, and surgical therapy was more invasive and risky when treating patients with systemic problems [2, 3].

It has been reported that one of the functions of PDL cells is to respond to environmental stresses, such as inflammatory cytokines and pathogens [4, 5]. Sun et al. reported that lipopolysaccharide (LPS) exposure induces the expression of cytokines in PDL cells, including tumor necrosis factor (TNF)-α, interleukin (IL)-1β, IL-6, and IL-8 [4]. Inflammatory cells as well as PDL cells regulate the periodontitis process [4–6], although the exact roles of different cell types require further clarification.

LPS is a macromolecule with a lipid element (lipid A) and a polysaccharide element [7]. LPS is located on the exterior membrane of gram-negative bacteria and stimulates powerful immune responses in animals. Indeed, the immune systems of animals have evolved to recognize LPS via toll-like receptors (TLRs) [8]. TLRs are a class of transmembrane proteins that contain numerous leucine-rich repeats and are composed of globular intracellular regions and extracellular domains [9]. Eleven different TLRs in humans have been identified [10]. Among them, TLR2 and 4 function as the principal innate sensors for the virulent factors of periodontopathic bacteria [11, 12]. LPS binding to TLR 2 and 4 triggers activation of the nuclear factor kappa B (NF-κB) signaling cascade, inducing the secretion of pro-inflammatory cytokines such as TNF-α and IL-1β [13]. Alterations to the number, position, and length of the acyl groups or monosaccharide groups in lipid A will result in alterations in the biologic effects [14]. Based on these mechanisms, LPS from different bacterial species may trigger different types of immune responses.

In the past few decades, low-level laser therapy (LLLT) has been widely employed in the treatment of wound healing, musculoskeletal pain, and chronic and acute inflammation [15, 16]. Studies have identified the benefits of using LLLT [17, 18]. For example, Bortone et al. reported that LLLT induced an anti-inflammatory effect through the regulation of pro-inflammatory mRNAs [17]. Obradovic et al. reported that low-level lasers have shown efficacy in reducing inflammation of periodontitis in patients with diabetes mellitus [18]. However, the precise mechanism by which LLLT regulates periodontal inflammation remains unknown.

Cyclic adenosine monophosphate (cAMP) functions as an important second messenger in many cell physiological processes, such as cell differentiation, proliferation, apoptosis, and inflammation [19]. cAMP is formed by the activation of adenylyl cyclase and is converted from adenosine triphosphate [19]. Also, cAMP was recently implicated in pro-inflammatory processes as it was shown to not only serve as a modulator of immune function but also as a regulator in response to LLLT signaling [20]. However, the potential relationship between cAMP and LLLT in affecting the LPS-induced inflammatory response in human periodontal ligament cells (hPDLCs) has not been investigated.

In this study, we investigated the anti-inflammatory effects of LLLT on hPDLCs that were exposed to LPS from *Porphryromonas gingivalis* (*P. gingivalis*) or *Escherichia coli* (*E. coli*) and aimed to determine the possible mechanism of action.

## Materials and methods

### Cell culture

The procedure for hPDL cell preparation was according to a modification of the method reported by D’Errico et al. [21]. Briefly, hPDL tissues were collected from the middle third of periodontally healthy and non-carious third molar roots extracted from healthy volunteers (20 to 40 years of age) during the course of orthodontic treatment. Periodontal ligament tissue was scraped from third molars, enzymatically digested by 2 mg/mL collagenase and 0.25% trypsin for 1 h at 37 °C. The cells were harvested by centrifugation for 10 min at 500×*g*. The supernatant was carefully aspirated, and cells were washed twice with Dulbecco’s modified Eagle’s medium (DMEM) containing 10% fetal bovine serum. The cells were expanded with DMEM supplemented with 10% fetal bovine serum, 100 mg/mL penicillin-G, 100 mg/mL kanamycin sulfate, and 0.3 mg/mL amphotericin B. The cultures were kept at 37 °C in a humidified incubator in the presence of 95% air and 5% CO_2_. The medium was changed every other day. The cells were used at passages 3 to 9.

### Chemical inhibitor and reagent treatments

LPS from *E. coli* and *P. gingivalis* was dissolved in 1× phosphate-buffered saline at a final concentration of 10 mg/mL and stored at 4 °C. The hPDLCs were treated with different concentrations of LPS (0, 10, 20, 50, and 100 μg/mL) from *P. gingivalis* and *E. coli* freshly diluted in culture medium. The adenylyl cyclase inhibitor SQ22536 (Sigma-Aldrich, St. Louis, MO, USA) was used to block the activity of adenylyl cyclase. SQ22536 was dissolved in dimethyl sulfoxide (DMSO), and the cells were treated with a dose of 100 μmol/L. Forskolin (50 μmol/L, Sigma-Aldrich, St. Louis, MO, USA), an adenylyl cyclase activator, was dissolved in DMSO.

### Low-level laser therapy

A gallium-aluminum-arsenide red laser with flat top beam profile (wavelength 660 nm diode laser, Transverse Industries Co., Ltd., Taipei, Taiwan) was used as the laser source. The output of the laser device was 70 mW. The distance between the bottom of the culture plate and the laser source was 3 cm, and the irradiated area was 3.8 cm^2^. The cells were irradiated once for 528 s in a continuous mode and received 8 J/cm^2^ of laser energy density in total. All irradiation experiments were performed on a clean bench at room temperature. The control groups were processed under the same conditions, except without laser irradiation.

### Cell viability

An MTT assay (3-[4, 5- dimethylthiazol-2-yl]-2, 5-diphenyltetrazolium bromide) was used to determine cell viability. The hPDLCs were seeded onto a 96-well plate. After exposure to LPS from *P. gingivalis* and *E. coli* for 24 h, the cells were incubated with 5 mg/mL MTT for 4 h. The reactions were measured using an enzyme-linked immunosorbent assay (ELISA) reader at 595 nm.

### Lactate dehydrogenase leakage

Lactate dehydrogenase (LDH) leakage was measured to quantify cytotoxicity with a Cytotoxicity Detection kit. The hPDLCs were seeded onto a 96-well plate and cultured with different concentrations of LPS from *P. gingivalis* and *E. coli* for 24 h. The LDH leakage was calculated according to the manufacturer’s guidelines. The absorbance was measured with an ELISA reader at 490 nm.

### Real-time reverse transcription-polymerase chain reaction (RT-PCR)

The total RNA from hPDLCs was extracted using TRIzol (Thermo Fisher Scientific, Waltham, MA, USA). cDNA reverse transcription was conducted with an RT system containing Moloney Murine Leukemia Virus reverse transcriptase. Quantitative real-time PCR was performed in a Bio-Rad CFX Connect real-time PCR detecting system, and the reactions were carried out in a mixture containing cDNA, primers for each gene, and iQ™ Syber-Green Supermix (Bio-Rad, Hercules, CA, USA). The primer pair sequences for the following human genes are listed in Table 1. The relative mRNA expression levels were analyzed with the comparative Ct method, where the amount of target is normalized to the housekeeping gene GAPDH.

**Table 1:** Primer sequences

Gene	Primer sequence		Accession number
IL-1β	Forward:	5′-AAACCTCTTCGAGGCACAAG-3′	NM_000576
	Reverse:	5′-GTTTAGGGCCATCAGCTTCA-3′	
TNF-α	Forward:	5′-CTCGAACCCCGAGTGACAAG-3′	NM_000594.3
	Reverse:	5′-TGAGGTACAGGCCCTCTGAT-3′	
IL-6	Forward:	5′-CCTGACCCAACCACAAATGC-3′	NM_000600.3
	Reverse:	5′-ATCTGAGGTGCCCATGCTAC-3′	
IL-8	Forward:	5′-CAGGAATTGAATGGGTTTGC-3′	NM_000584.3
	Reverse:	5′-AAACCAAGGCACAGTGGAAC-3′	
GAPDH	Forward:	5′-CAATGACCCCTTCATTGACC-3′	NM_002046
	Reverse:	5′-TTGATTTTGGAGGGATCTCG-3′	

### Intracellular cyclic adenosine monophosphate levels

ELISA kits for the detection of cAMP were obtained from Enzo (Farmingdale, NY, USA). All of the procedures were based on the manufacturer’s instructions, and the optical density was measured at a wavelength of 405 nm.

### Dual-luciferase assay

We cultured 2 × 10^4^ hPDLCs in a 48-well plate and co-transfected the cells with the pGL 4.32 [luc2P/NF-κB-RE/Hygro] vector (250 ng) and pTK-Renilla vector (50 ng) using Lipofectamine 2000 (Thermo Fisher Scientific, Waltham, MA, USA). The transfection procedure was performed according to the manufacturer’s instructions. Twenty-four hours after transfection, the cells were treated with LPS, LLLT, and SQ22536 for an additional 6 h. The luciferase activity was measured using a dual-luciferase reporter assay system (Promega, Madison, WI, USA) with an Anthos Lucy3 microplate luminometer (AnthosLabtec Instruments, Austria).

### Statistical analysis

All the data were collected from three to four independent experiments. Data were analyzed with the statistical software SPSS/Win version 17.0 and expressed as the mean ± standard deviation. For statistical analysis, normality was first tested using the Shapiro-Wilk test. For normally distributed data, one-way analysis of variance was used to test for statistical differences followed by the post hoc Tukey’s test. If the data failed the normality test, the nonparametric Kruskall-Wallis one-way analysis of variance was used to test for statistical differences with Dunn’s multiple comparison test. A *p* value of 0.05 was considered statistically significant.

## Results

### The viability and cytotoxicty of LPS-challenged hPDLCs

**Fig. 1:**
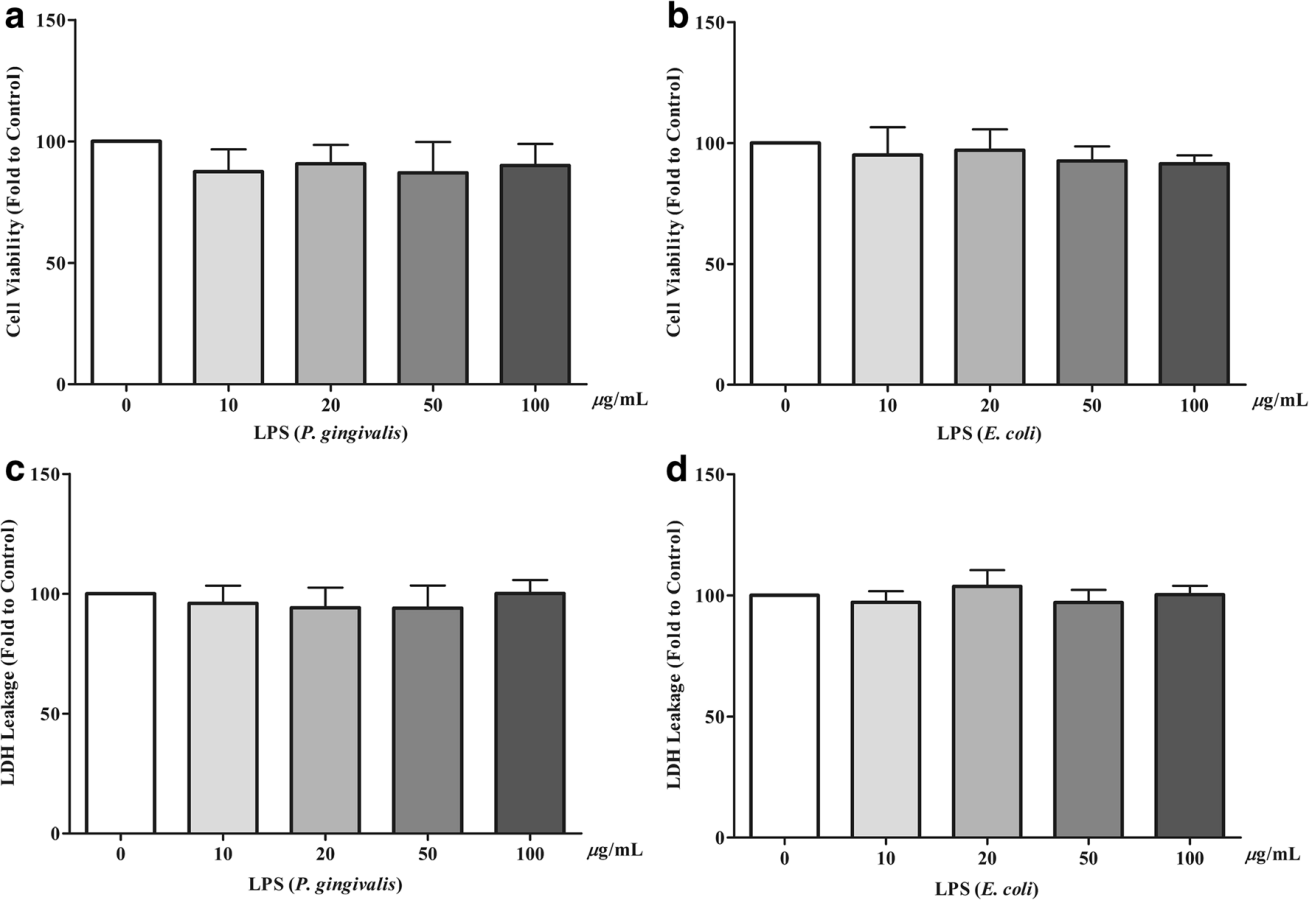
LPS did not induce cytotoxic effects nor reduce the viability of hPDLCs. The hPDLCs were treated with LPS from *P. gingivalis* or *E. coli* doses of 0 (control), 10, 20, 50, or 100 μg/mL. MTT assays (**a**, **b**) and LDH leakage analysis (**c**, **d**) were used to evaluate cell viability and cytotoxicity. The data are shown as the mean ± SD. *N* = 3 (*N* numbers of independent experiments). There were no significant differences between the groups

To evaluate the influence of LPS from *P. gingivalis* and *E. coli* on the viability of hPDLCs, an MTT test was conducted. The MTT assay showed that the viability of hPDLCs was not reduced following treatment with *P. gingivalis* or *E. coli* LPS at 24 h (Fig. 1a, b). The cytotoxic effects of LPS on hPDLCs were also measured with the LDH leakage assay. After normalizing to the control group, the activities of LDH leakage showed no differences between the control and LPS-treated groups at 24 h (Fig. 1c, d). These results indicated that LPS was not cytotoxic and did not reduce the viability of hPDLCs in the short time intervals. All subsequent experiments were analyzed in 24-h increments.

### The expression patterns of inflammatory cytokines in LPS-challenged hPDLCs

**Fig. 2:**
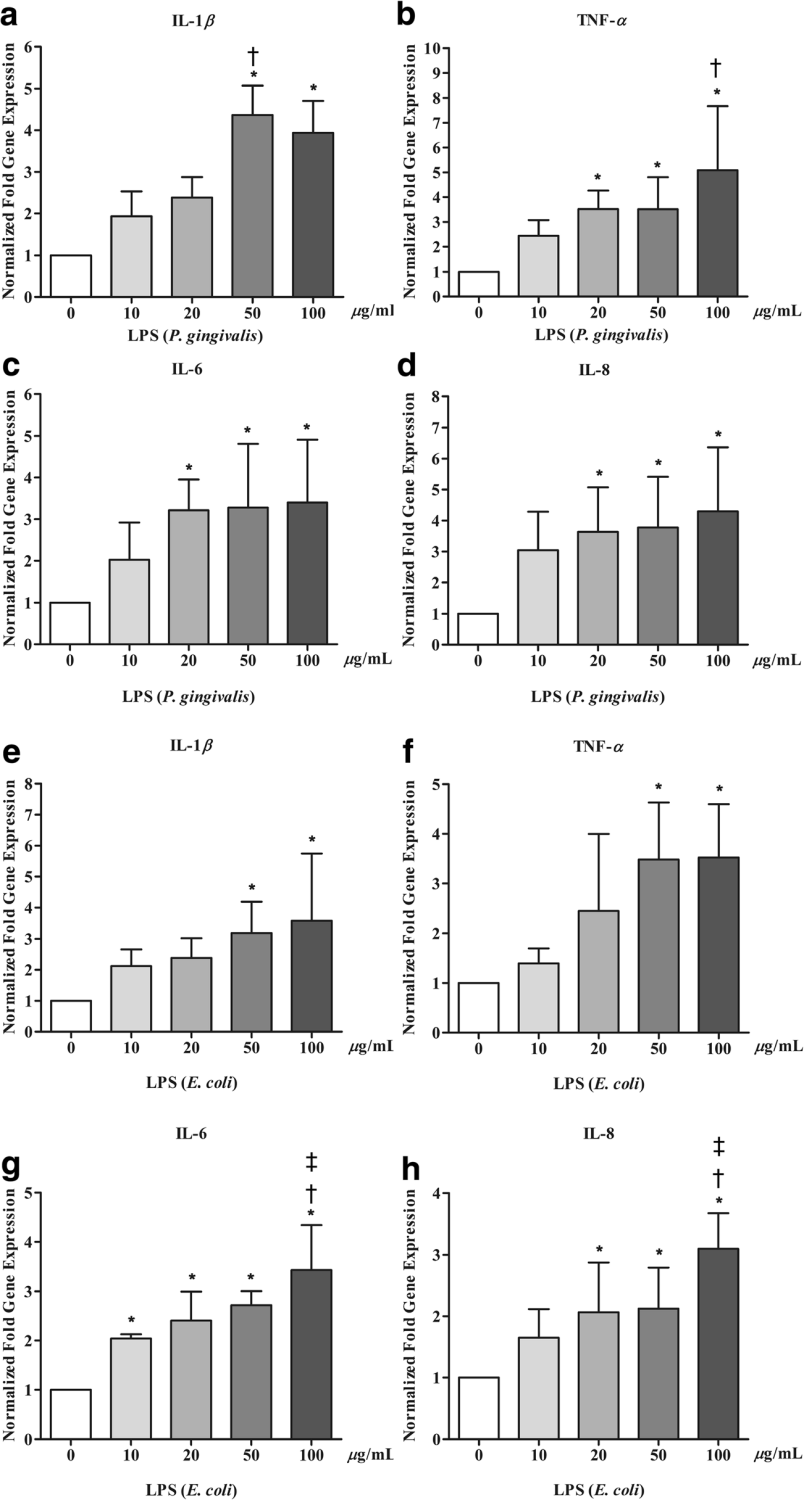
hPDLCs stimulated by LPS expressed inflammatory genes. LPS from *P. gingivalis* or *E. coli* increased the mRNA expression of IL-1β, TNF-α, IL-6, and IL-8. **a**–**d** Cells treated with LPS from *P.gingivalis*. **e**–**h** Cells treated with LPS from *E. coli*. The data are shown as the mean ± SD. *N* = 4 (*N* numbers of independent experiments). The following statistical levels were applied: *p < 0.05 compared with the 0 μg/mL group (control); †*p* < 0.05 compared with the 10 μg/mL group; ‡*p* < 0.05 compared with the 20 μg/mL group

The hPDLCs were treated with different concentrations of LPS from *P. gingivalis* or *E. coli* for 24 h. Then, q-PCR was conducted to assess the gene expression of pro-inflammatory cytokines including IL-1β, TNF-α, IL-6, and IL-8. The results showed that LPS from *P. gingivalis* or *E. coli* enhanced the mRNA expression of TNF-α, IL-1β, IL-6, and IL-8 in a dose-dependent manner with increasing LPS concentrations. LPS from *P. gingivalis* induced a 2- to 4-fold elevation of mRNA expression at a lower dose (20 μg/mL), while LPS from *E. coli* had the same effect at a higher dose (50 μg/mL) (Fig. 2). At doses higher than 50 μg/mL, the expression levels of most pro-inflammatory cytokine genes were observed to plateau (Fig. 2a, c–f).

### The anti-inflammation effect of LLLT on hPDLCs

**Fig. 3:**
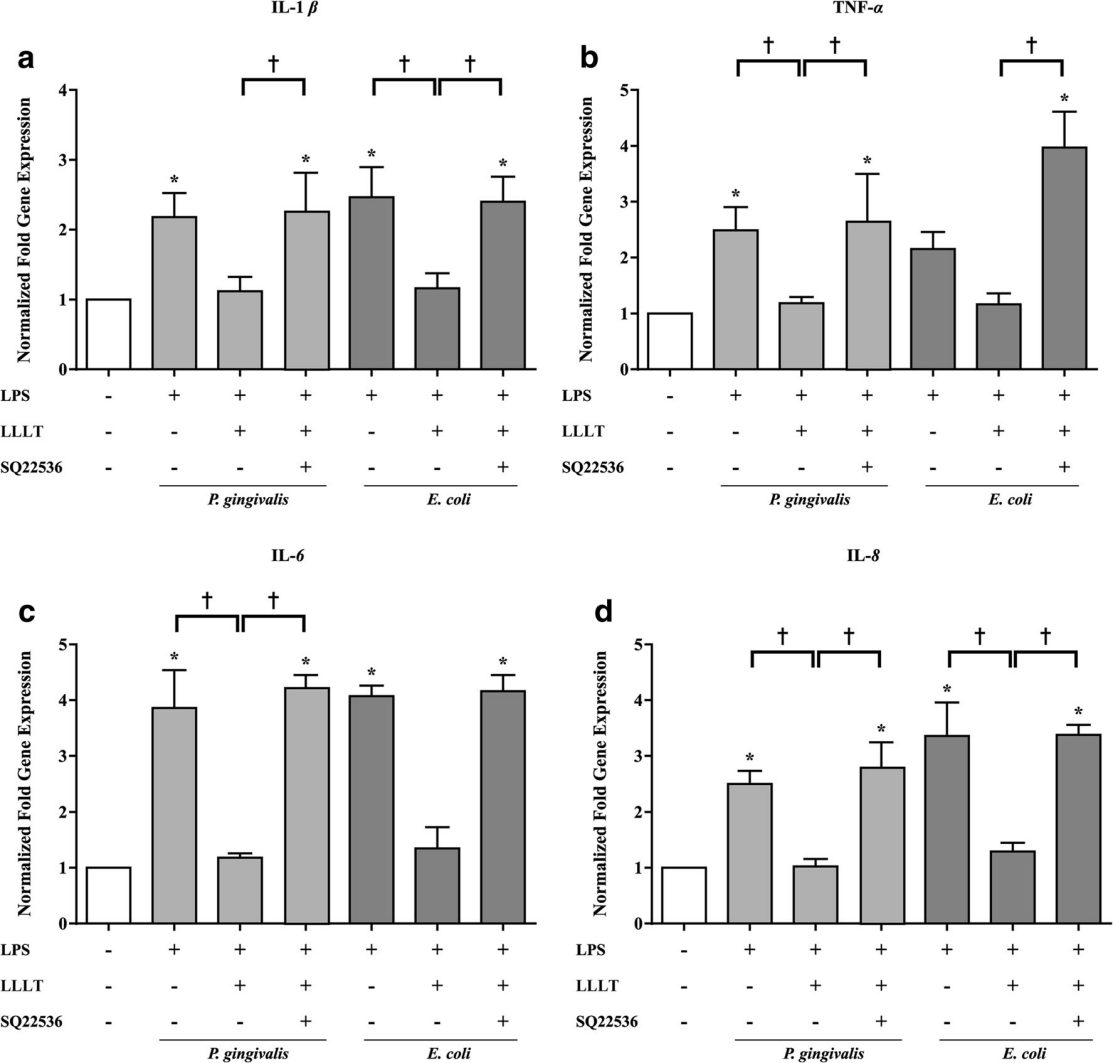
A cAMP inhibitor (SQ22536) hindered the anti-inflammatory effect of LLLT on inflammation induced by LPS of *P. gingivalis* and *E. coli*. Real-time RT-PCR was performed to measure the mRNA levels of **a** IL-1β, **b** TNF-α, **c** IL-6, and **d** IL-8. The results were analyzed with the 2^−ΔCT^ method based on the control. The data are shown as the mean ± SD. *N* = 4 (*N* numbers of independent experiments). The following statistical levels were applied: *p < 0.05 compared with the control group and †*p* < 0.05

The hPDLCs were treated with or without LPS from *P. gingivalis* (20 *μ*g/mL) or *E. coli* (50 μg/mL), which was immediately followed by laser irradiation at an energy density of 8 J/cm^2^ to determine the effect of LLLT on the LPS-induced inflammatory response. Twenty-four hours following the exposure of hPDLCs to LPS from *P. gingivalis* or *E. coli*, there was an elevated level of inflammatory cytokine mRNA expression in the non-irradiated group. The group treated with LPS and LLLT showed obvious decreases in cytokine mRNAs compared to the LPS-only group (Fig. 3).

To elucidate whether LLLT inhibited inflammatory cytokine mRNA expression by regulating cAMP, we treated hPDLCs with SQ22536, a pharmacological inhibitor of cAMP, and then treated with LPS and LLLT. In this group, the level of pro-inflammatory cytokine mRNA expression was not reduced by LLLT (Fig. 3).

**Fig. 4:**
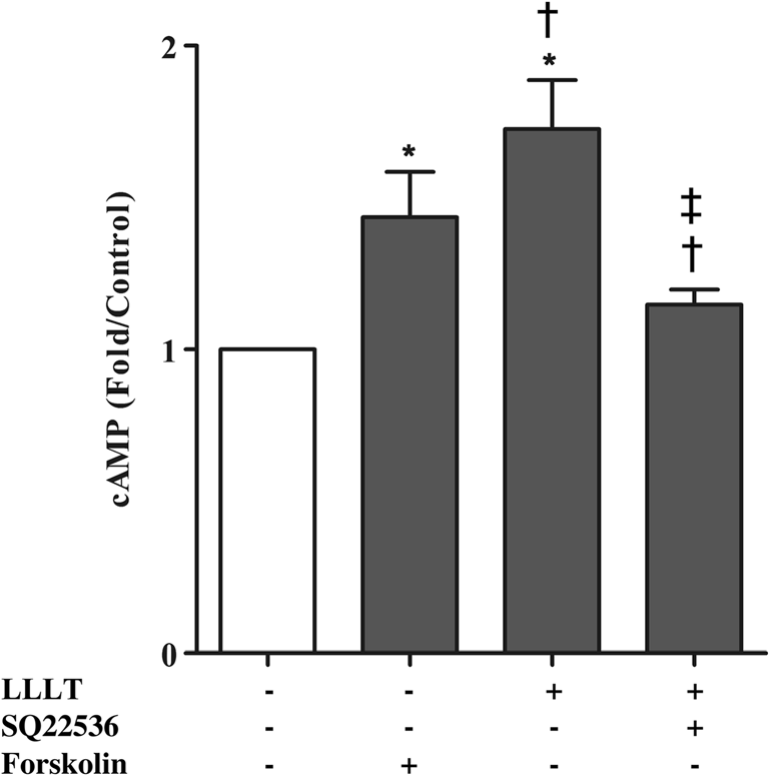
LLLT increased the intracellular cAMP levels. Intracellular cAMP levels were measured by ELISA in hPDLCs treated with LLLT, cAMP inhibitor (SQ22536), or forskolin. The data are shown as the mean ± SD. *N* = 4 (*N* numbers of independent experiments). The following statistical levels were applied: *p < 0.05 compared with the control group; †*p* < 0.05 compared with the forskolin-only group; ‡*p* < 0.05 compared with the LLLT-only group

**Fig. 5:**
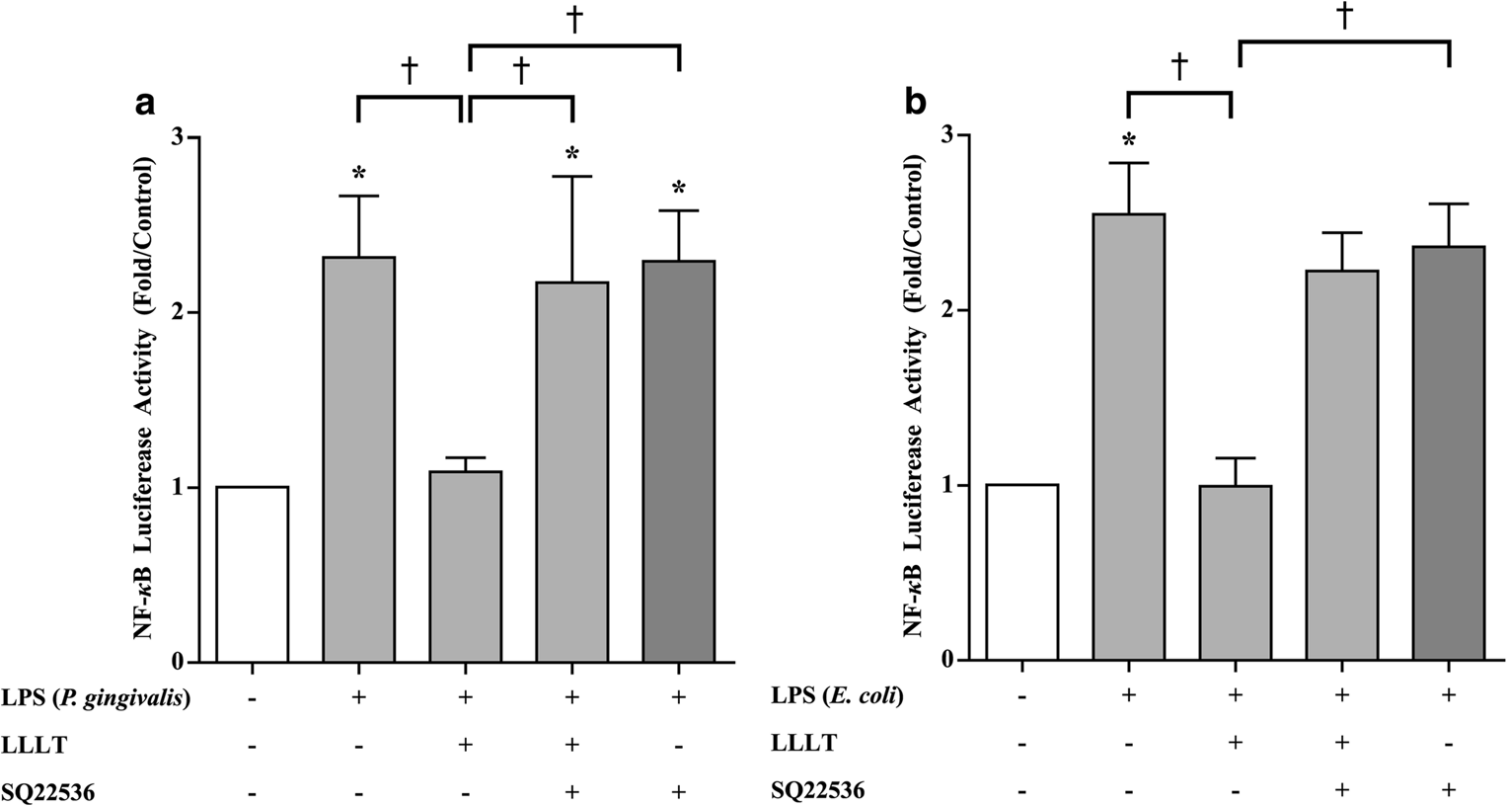
LLLT reduced NF-κB transcriptional activity. The NF-κB luciferase activity was elevated by LPS and reduced by LLLT. With the addition of the cAMP inhibitor (SQ22536), the NF-κB activity in LPS-stimulated hPDLCs was not reduced by LLLT: **a** LPS from *P. gingivalis*; **b** LPS from *E. coli.* The data are shown as the mean ± SD. *N* = 4 (*N* numbers of independent experiments). The following statistical levels were applied: *p < 0.05 compared with control group; †*p* < 0.05

Next, an ELISA was conducted to examine the cAMP levels associated with LLLT in hPDLCs. When hPDLCs were treated with the adenylyl cyclase activator forskolin or irradiated with the low-level laser, the level of cAMP increased and showed a significant difference compared to the control group. However, when hPDLCs were treated with SQ22536 and irradiated with the low-level laser, the level of cAMP was not elevated and showed a significantly lower level when compared to the LLLT or forskolin-only groups (Fig. 4). These results indicated that LLLT may reduce inflammation through the regulation of cAMP. We further investigated whether LLLT affected the transcriptional activity of NF-κB, a crucial inflammatory transcription factor, using luciferase reporter assays. When hPDLCs were treated with LPS from *P. gingivalis* (20 μg/mL) or *E. coli* (50 μg/mL), the level of NF-κB was increased and showed a significant difference compared to the control group (Fig. 5). When hPDLCs were treated with LPS and irradiated with the low-level laser, the level of NF-κB showed no significant difference compared to the control group. With the addition of SQ22536, the irradiated LPS-stimulated hPDLCs showed a similar NF-κB level as the LPS-only group and a major difference when compared to the control and laser-irradiated LPS-stimulated groups (Fig. 5). These results suggested that LLLT regulated NF-κB transcriptional activity by affecting the cAMP level.

## Discussion

The hPDLCs are human fibroblasts with multifunctional tissue that provide physical, formative and remodeling, nutritional, and sensory functions [22]. In the present study, the hPDLCs were treated with different concentrations of LPS from *P. gingivalis* or *E. coli*. Our data showed that LPS from both *P. gingivalis* and *E. coli* induced pro-inflammatory cytokine (IL-1β, TNF-α, IL-6, IL-8) mRNA expressions in a dose-dependent manner. However, *P. gingivalis*-treated cells responded in a dose-dependent manner at lower doses when compared to *E. coli*-treated cells (Fig. 2). The different levels of induced inflammation from the two sources may be attributed to molecular differences in the lipid components. The lipid A from *P. gingivalis* LPS is a monophosphate type lacking a phosphate group in the 4′ position that does not contain a tetradecanoic acid, but rather long-chain fatty acids made up of only acyloxyl groups [23]. It is believed that LPS from *P. gingivalis* is recognized mainly by TLR2, while LPS from *E. coli* is recognized mainly by TLR4 [23–26]. Uehara et al. also reported that human gingival fibroblasts showed more robust mRNA expression of pro-inflammatory cytokines following TLR2 ligation (*Mycoplasma*-type diacyl lipopeptide) compared to TLR4 ligation (*E. coli*-type lipid A) [27]. These results indicate that different sources of LPS might induce inflammation through different mechanisms.

In recent decades, many studies have shown that LLLT reduces inflammatory reactions both in vitro and in vivo. Correa et al. induced periodontitis in mice with LPS and found that LLLT (GaAs laser, 904 nm) diminished inflammatory cell migration in a dose-dependent manner, with an energy dose of 3 J/cm^2^ identified as the most effective dose [28]. Pires et al. reported a model of collagenase-induced tendinitis and demonstrated that LLLT (780 nm), at an energy dose of 7.7 J/cm^2^, suppressed the expression of IL-6 [29]. Boschi et al. reported that LLLT (InGaAlP laser, 660 nm) significantly reduced the expression of IL-6 and TNF-α [30]. According to previous studies conducted in our lab [31], LLLT (GaAlAs laser, 660 nm) showed the most effective suppression of inflammation at the optimal dose of 8 J/cm^2^, which was used in this study. In the current study, we noted that LPS-challenged hPDLCs showed similar results to the aforementioned studies, suggesting that LLLT significantly suppressed the mRNA expression of pro-inflammatory cytokines (IL-1β, TNF-α, IL-6, IL-8), leading us to conclude that LLLT has an anti-inflammatory effect in hPDLCs (Fig. 3). In addition, we noticed that the anti-inflammatory effect of LLLT was neutralized when the cAMP inhibitor SQ22536 was used (Fig. 3). We also observed that the level of cAMP from hPDLCs was elevated by both forskolin (cAMP promoter) and LLLT but decreased when SQ22536 (cAMP inhibitor) was added (Fig. 4), consistent with previous study that the level of cAMP increased by approximately 3- to 4-fold following LLLT treatment of human adipose-derived stem cells [31]. This finding suggests that LLLT can act to stimulate the level of cAMP. Other studies have shown that the elevation of intracellular cAMP levels inhibited the transcriptional activity of NF-κB, which is a crucial transcription factor in the regulation of inflammation [32, 33]. Possible mechanisms that regulate of NF-κB activity include the ability of cAMP to manage IκB degradation and IKK activity as well as to change the composition of NF-κB dimers and thereby block transcription [34]. Indeed, we observed that LLLT significantly inhibited the transcriptional activity of NF-κB in our previous study of LPS-stimulated human adipose-derived stem cells [31]. In the present study, we treated LPS-stimulated hPDLCs with LLLT and the adenylyl cyclase inhibitor SQ22536 and observed that the inhibitory effect of LLLT on NF-κB transcriptional activity was significantly reduced (Fig. 5). Aimbire et al. [35] also reported results similar to ours, showing that a low-level laser (660 nm) at an energy dose of 7.5 J/cm^2^ inhibited NF-κB transcriptional activity and further reduced apoptotic gene expression.

Exploring LLLT’s role in regulating the induction of pro-inflammatory cytokine in periodontal pathology is important as it may lead to novel therapeutic approaches for periodontitis. This study has demonstrated that LLLT inhibits inflammation, induced by LPS from *E. coli* and *P. gingivalis*, through the cAMP/NF-ĸB signaling pathway in hPDLCs. Future research into the detailed regulation of LLLT on cAMP may be of great value in improving periodontal therapy.
